# Anesthetic Neuroprotection Based on Ion-Channel-Modulating Drugs in Acute Traumatic Brain Injury

**DOI:** 10.7759/cureus.104943

**Published:** 2026-03-09

**Authors:** Elia L Zamora, Diego Alvarez Ramirez, Christian Andrés Soto Cordero, Ronald Chavarría, Sabrina Montoya

**Affiliations:** 1 Emergency Medicine, Hospital Monseñor Sanabria Martínez, Puntarenas, CRI

**Keywords:** anesthetic agents, calcium dysregulation, ion-channel modulation, neuroprotection, secondary brain injury, traumatic brain injury

## Abstract

Traumatic brain injury (TBI) remains a major cause of disability and mortality. Secondary brain injury, driven by ionic imbalance, excitotoxicity, mitochondrial dysfunction, and inflammation, plays a critical role in neurological deterioration. Ion-channel dysregulation, particularly calcium overload and sustained membrane depolarization, represents a key therapeutic target.

This narrative review synthesizes recent evidence (2020-2025) on anesthetic agents that modulate ion channels in acute TBI. Dexmedetomidine, ketamine, propofol, volatile anesthetics, nimodipine, magnesium, lidocaine, and xenon exert neuroprotective effects through N-methyl-D-aspartate (NMDA) antagonism, sodium- and calcium-channel modulation, enhancement of inhibitory signaling, and stabilization of neuronal excitability. Preclinical studies demonstrate reduced apoptosis, preserved mitochondrial function, and attenuation of excitotoxic injury.

Clinical translation remains limited by heterogeneity in injury patterns and study design. Nonetheless, a mechanism-guided approach to anesthetic selection, aligned with predominant secondary injury pathways and hemodynamic priorities, may enhance neuroprotective strategies in both neuro-intensive care unit (ICU) and intraoperative settings. Further high-quality clinical trials are required to define the optimal role of ion-channel-modulating anesthetics in TBI management.

## Introduction and background

Traumatic brain injury (TBI) remains a leading cause of disability and mortality worldwide, resulting in long-term cognitive, behavioral, and motor impairments. While the primary mechanical insult is immediate and largely irreversible, much of the neurological deterioration occurs during the subsequent phase of secondary brain injury. This delayed cascade, characterized by inflammation, oxidative stress, mitochondrial dysfunction, and apoptotic signaling, offers a critical window for therapeutic intervention [1].

Among the mechanisms driving secondary injury, ionic imbalance plays a central role. Dysregulation of calcium, sodium, and potassium homeostasis disrupts neuronal membrane stability and intracellular signaling. In particular, excessive calcium influx contributes to mitochondrial failure, activation of cell-death pathways, and amplification of excitotoxic damage, positioning ion-channel dysfunction as a key pathophysiological driver and potential therapeutic target [2].

Within this context, anesthetic agents capable of modulating ion channels have gained attention not only for their sedative properties but also for their potential neuroprotective effects. Drugs such as dexmedetomidine, nimodipine, and dantrolene influence calcium signaling and inflammatory pathways, while other anesthetics act through N-methyl-D-aspartate (NMDA) antagonism, sodium-channel inhibition, or enhancement of inhibitory neurotransmission [2-4]. However, despite promising experimental findings, translation into consistent clinical benefit remains uncertain due to injury heterogeneity and variability in patient physiology.

This review aims to integrate current evidence on ion-channel-modulating anesthetic agents and propose a mechanism-based framework to inform anesthetic and neurocritical management in acute TBI. By linking molecular targets to clinical decision-making, we seek to clarify the potential role of these agents in mitigating secondary brain injury.

## Review

Methods

This manuscript was conducted as a structured narrative review aimed at synthesizing contemporary evidence on anesthetic neuroprotection mediated through ion-channel-modulating agents in acute TBI. The objective was to integrate molecular mechanisms, translational data, and clinical implications within a neurocritical care and anesthetic management framework.

A targeted literature search was performed in PubMed, Scopus, and ScienceDirect due to their broad coverage of biomedical and neuroscientific research. Peer-reviewed articles published between January 2020 and December 2025 in English or Spanish were considered. The search strategy combined Medical Subject Headings (MeSH) and free-text terms using Boolean operators as follows: (“Neuroprotection” OR “Secondary Brain Injury”) AND (“Ion Channel Modulation” OR “Calcium Channels” OR “Sodium Channels”) AND (“Traumatic Brain Injury”) AND (“Anesthetic Agents” OR “Neuroanesthesia”).

Titles and abstracts were screened for relevance, followed by full-text review when appropriate. Studies were excluded if they (1) were duplicate publications, (2) were not peer-reviewed, (3) did not involve TBI or related secondary injury mechanisms, (4) did not evaluate anesthetic or ion-channel-modulating agents, or (5) lacked clinically or mechanistically relevant outcome measures. Purely experimental studies confined to cellular or animal models without clear translational implications were excluded.

Eligible sources included randomized controlled trials, observational clinical studies, systematic reviews, meta-analyses, translational experimental research with defined clinical relevance, and consensus statements from recognized scientific societies. Given the narrative design, no formal risk-of-bias tool or quantitative synthesis was performed. Instead, methodological quality was appraised qualitatively based on study design, internal validity, sample characteristics, consistency of findings, and translational applicability. When conflicting evidence was identified, greater interpretative weight was given to higher-level clinical evidence and guideline-supported data.

Pathophysiology of acute TBI

TBI generates two distinct phases of damage, each with different mechanisms and therapeutic implications. The primary injury occurs at the moment of impact and encompasses mechanical events such as contusions, lacerations, and diffuse axonal injury, all of which result from the immediate physical disruption of brain tissue. This phase is largely irreversible. In contrast, secondary injury develops over hours to days after the initial trauma and involves a series of biochemical and cellular processes including inflammation, excitotoxicity, oxidative stress, and apoptosis that progressively worsen the initial damage. Because these mechanisms evolve over time, they offer critical opportunities for therapeutic intervention aimed at limiting further neuronal loss [5,6].

A central component of secondary injury is early ionic disruption, which affects calcium, sodium, potassium, and chloride homeostasis. Disruption of intracellular calcium balance is particularly harmful, as excessive Ca²⁺ influx through voltage-gated calcium channels and dysfunctional calcium pumps initiates a cascade of destructive intracellular processes that ultimately lead to neuronal death. Alterations in sodium and potassium levels specifically increased Na⁺ influx and K⁺ efflux promote sustained depolarization and contribute to excitotoxic mechanisms. Meanwhile, chloride imbalance exacerbates cellular swelling and promotes the development of cytotoxic edema [7].

**Figure 1:**
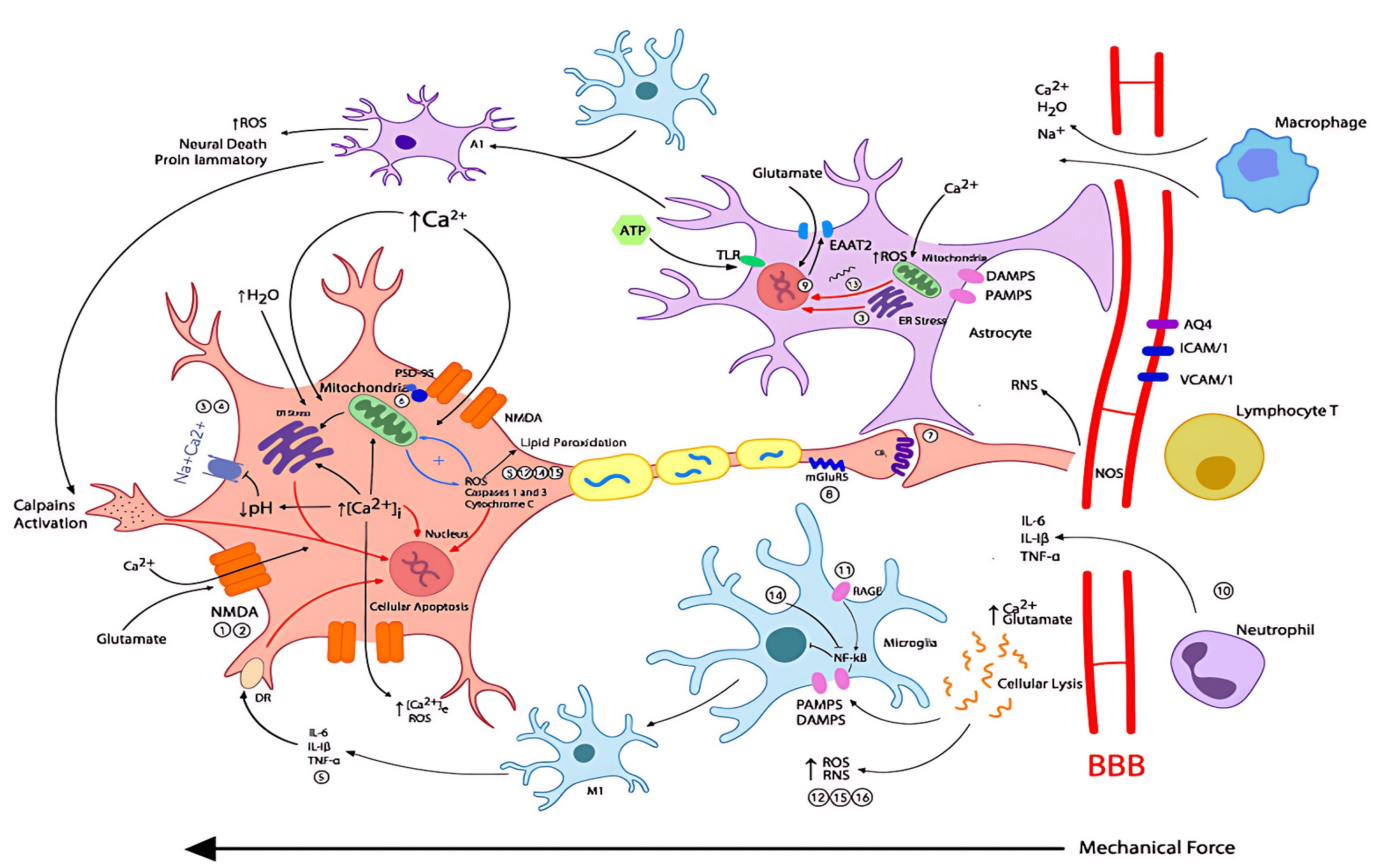
Pathophysiological cascade leading to excitotoxic neuronal injury after traumatic brain injury BBB disruption leads to ionic imbalance, immune cell infiltration, astrocyte and microglial activation, and glutamate accumulation. Excessive activation of NMDA receptors increases intracellular Ca²⁺, triggering mitochondrial dysfunction, oxidative stress, and apoptotic pathways. A1: astrocyte phenotype A1 (reactive astrocyte type A1); AQP4: aquaporin 4; ATP: adenosine triphosphate; BBB: blood-brain barrier; Ca²⁺: calcium ion; Calpains: calcium-dependent proteases; DAMPs: damage-associated molecular patterns; DR: death receptor; EAAT2: excitatory amino acid transporter 2; ER Stress: endoplasmic reticulum stress; H₂O: water; ICAM-1: intercellular adhesion molecule 1; IL-1β: interleukin 1 beta; IL-6: interleukin 6; mGluRs: metabotropic glutamate receptors; M1: microglial phenotype M1 (pro-inflammatory microglia); Na⁺: sodium ion; Na⁺/Ca²⁺: sodium-calcium exchanger; NF-κB: nuclear factor kappa B; NMDA: N-methyl-D-aspartate receptor; nNOS: neuronal nitric oxide synthase; PAMPs: pathogen-associated molecular patterns; PSD-95: postsynaptic density protein 95; RAGE: receptor for advanced glycation end products; RNS: reactive nitrogen species; ROS: reactive oxygen species; TLR: Toll-like receptor; TNF-α: tumor necrosis factor alpha; VCAM-1: vascular cell adhesion molecule 1 Figure Source: Baracaldo-Santamaría et al. [8]; reproduced under the Creative Commons Attribution License (CC BY 4.0 Deed)

These ionic disturbances are closely linked to glutamate-mediated excitotoxicity, another major contributor to secondary injury. Excessive extracellular glutamate overstimulates NMDA and α-amino-3-hydroxy-5-methyl-4-isoxazolepropionic acid (AMPA) receptors, driving further Ca²⁺ entry and amplifying neuronal damage [8,9]. Overactivation of NMDA receptors is especially detrimental because it accelerates calcium overload and triggers pathways that culminate in cell death [10]. Figure 1 summarizes the key mechanisms of secondary brain injury, integrating ionic imbalance, glutamate-mediated excitotoxicity, mitochondrial dysfunction, oxidative stress, neuroinflammation, and blood-brain barrier disruption. It highlights the central role of calcium influx and NMDA receptor overactivation in amplifying neuronal damage and provides a visual framework for the interconnected processes described above.

Mitochondrial dysfunction represents an additional hallmark of secondary injury. Following TBI, mitochondria exhibit impaired energy production and generate excessive reactive oxygen species, which cause oxidative damage to lipids, proteins, and DNA. The resulting free radical accumulation intensifies oxidative stress and promotes apoptotic cell death [11].

**Figure 2:**
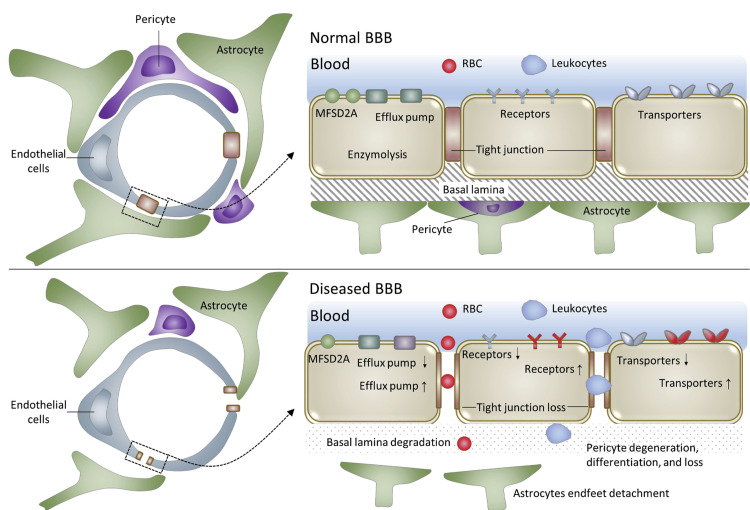
BBB structure under normal physiological conditions (top) and disrupted barrier during neurological disease or injury (bottom) BBB: blood-brain barrier Figure Source: Han and Jiang [12]; reproduced under the CC BY-NC-ND License

Inflammation further compounds neuronal injury. Activation of microglia and astrocytes leads to the release of pro-inflammatory cytokines that amplify tissue damage and contribute to widespread neuroinflammatory responses [5,6]. These inflammatory processes can compromise the integrity of the blood-brain barrier, facilitating the formation of cerebral edema, which increases intracranial pressure (ICP) and contributes to additional neurological deterioration [5]. As shown in Figure 2, disruption of the BBB plays a central role in the development of cerebral edema and secondary neuronal injury.

TBI can impair cerebral autoregulation, reducing the brain's ability to maintain stable blood flow and oxygen delivery despite changes in systemic pressure. This impairment predisposes damaged regions to ischemia and exacerbates the progression of secondary injury, reinforcing the complexity and interconnectedness of these pathophysiological mechanisms [5].

Ion channels as therapeutic targets

Ion-channel dysfunction is a central contributor to secondary brain injury following TBI. Disruptions in sodium, calcium, potassium, and glutamate receptor-mediated signaling promote sustained depolarization, intracellular calcium overload, and activation of cell-death pathways, making these channels rational therapeutic targets [1].

Voltage-gated sodium channels are essential for action potential initiation and propagation and therefore play a key role in neuronal excitability [13]. After TBI, persistent activation of Nav channels contributes to pathological depolarization and metabolic stress. Pharmacologic modulation of these channels may reduce hyperexcitability; for example, sevoflurane has been shown to attenuate abnormal depolarization and confer neuronal protection in experimental models [13,14].

Calcium channels, including L-type, N-type, and P/Q-type subtypes, regulate intracellular Ca²⁺ levels under physiological conditions. Following trauma, excessive calcium influx through these channels contributes to intracellular Ca²⁺ overload, mitochondrial dysfunction, and activation of apoptotic cascades [2,15]. Nimodipine, an L-type calcium channel blocker, has demonstrated the ability to reduce calcium overload and mitigate neuronal injury in the post-traumatic period [2].

Potassium channels, including Kv, KATP, and Kir families, play a stabilizing role in membrane potential regulation. Their activation promotes neuronal hyperpolarization, limiting excessive depolarization and reducing susceptibility to excitotoxic injury [16].

NMDA and AMPA receptors are central mediators of glutamate-driven excitotoxicity, a major mechanism of neuronal injury in TBI. Excessive activation enhances Ca²⁺ entry and accelerates downstream cell-death pathways. Pharmacological modulation of these receptors has been shown to reduce excitotoxic damage and improve outcomes in experimental models [17].

Additionally, transient receptor potential (TRP) channels, particularly TRPV1, TRPA1, and TRPM8, participate in nociceptive signaling and contribute to inflammatory responses and edema formation after TBI. Modulating these channels may attenuate neuroinflammation and limit edema-related complications [18].

Collectively, these findings underscore the therapeutic relevance of ion-channel modulation in secondary brain injury. Targeting sodium, calcium, potassium, glutamate, and TRP channels may reduce excitotoxicity and stabilize neuronal activity. Agents such as nimodipine and sevoflurane have shown promising effects in preclinical studies [2,13]. Nonetheless, translating these mechanistic benefits into consistent clinical outcomes remains challenging, highlighting the need for continued translational research in this field [1].

Anesthetic drugs with neuroprotective ion-channel modulation

Propofol exerts neuroprotective effects primarily through the potentiation of GABA_A receptors, enhancing inhibitory neurotransmission and reducing neuronal excitability [18]. By strengthening inhibitory tone, it indirectly limits pathological calcium influx and attenuates Ca²⁺-mediated injury pathways. In addition to its neuronal actions, propofol reduces cerebral metabolic demand and modulates cerebral blood flow, contributing to improved oxygen supply-demand balance during anesthesia [3]. These properties support its use in neurocritical care; however, concerns remain in vulnerable populations, particularly infants, where exposure has been associated with potential neurodevelopmental consequences [19,20].

Ketamine provides neuroprotection through the antagonism of NMDA receptors, thereby reducing glutamate-mediated excitotoxicity, a central mechanism in secondary brain injury [21]. By limiting NMDA receptor overactivation, ketamine decreases pathological calcium influx and downstream apoptotic signaling. Both experimental and clinical studies support its ability to attenuate excitotoxic damage, reinforcing its therapeutic relevance in acute neuronal injury contexts [22].

Barbiturates such as thiopental and pentobarbital offer neuroprotective effects through sodium-channel blockade and profound suppression of cerebral metabolic activity. By reducing neuronal excitability and lowering cerebral metabolic rate, they may protect against ischemic injury in selected clinical situations [20].

Volatile anesthetics, including isoflurane and sevoflurane, influence ion-channel activity and cerebral metabolism through distinct mechanisms. Isoflurane activates TREK-1 channels by disrupting phospholipase D2 signaling, reducing synaptic transmission and cerebral metabolic rate of oxygen consumption (CMRO₂) [23]. Experimental data suggest that isoflurane preserves neurovascular coupling and mitochondrial function under certain conditions [24]. Sevoflurane similarly decreases synaptic transmission and CMRO₂ while appearing to better preserve mitochondrial integrity compared with other anesthetic agents [24]. Its modulation of neuronal network activity contributes to proportional reductions in energy metabolism, which may be advantageous in injured brain tissue.

Xenon confers neuroprotection through selective NMDA receptor antagonism, inhibiting excitatory glutamatergic signaling while maintaining favorable hemodynamic stability [25]. This combination of anti-excitotoxic and cardiovascular properties supports its potential role in neuroprotective anesthetic strategies, particularly in ischemia-reperfusion settings.

Intravenous lidocaine exerts neuroprotective effects via the blockade of voltage-gated sodium channels. By inhibiting Nav channel activity, it reduces neuronal excitability and glutamate release, thereby attenuating excitotoxic cascades [26,27]. In addition to these neuroprotective properties, systemic lidocaine provides analgesic, anti-inflammatory, and immunomodulatory effects that may be beneficial in perioperative and critical care contexts.

Magnesium sulfate contributes to neuroprotection as a physiological NMDA receptor blocker. By limiting excessive calcium influx into neurons, it reduces glutamate-mediated excitotoxicity and modulates neuroinflammatory responses [28,29]. Furthermore, magnesium supports cerebral hemodynamics, reinforcing its potential role in mitigating secondary injury [3].

Calcium-channel blockers such as nimodipine provide neuroprotective benefits by stabilizing intracellular calcium levels and improving cerebrovascular regulation. By reducing calcium overload and preventing vasospasm, nimodipine supports maintenance of cerebral blood flow and oxygen delivery, mechanisms that are essential in preventing secondary ischemic injury [3,30].

Experimental and clinical evidence

Experimental models of TBI provide important insight into the neuroprotective potential of ion-channel-modulating agents. Sevoflurane has been shown to reduce neuronal apoptosis and improve neurological outcomes through the modulation of the p38-MAPK signaling pathway via the EZH2/KLF4 axis, resulting in decreased blood-brain barrier permeability and reduced brain water content [31]. Xenon administration in rat models has demonstrated improved locomotor performance, reduced lesion volume, and modulation of neuroinflammatory responses, supporting its potential as a neuroprotective intervention [32]. Similarly, memantine, an NMDA receptor antagonist, has inhibited cortical spreading depolarizations and improved neurovascular function, thereby limiting neurological deterioration in experimental settings [33]. Nimodipine has also demonstrated protective effects by blocking histone-induced calcium overload, reducing neuronal apoptosis, and enhancing functional recovery [2]. Collectively, these findings reinforce the mechanistic rationale for targeting ion channels in secondary brain injury.

Clinical data, although more limited, suggest potential translational relevance for selected agents. Trials involving ketamine indicate that NMDA antagonism may influence stress-related pathways and synaptic plasticity, though its impact on synaptic density and microglial activation warrants further investigation. While detailed randomized data for barbiturates and volatile anesthetics are less robust in the recent literature cited, their established use in TBI management reflects their capacity to reduce neuronal excitability and metabolic demand through ion-channel modulation [34].

Emerging pharmacological approaches continue to expand this landscape. Xenon's favorable experimental profile, together with its hemodynamic stability, positions it as a promising candidate for future clinical evaluation [32]. Novel compounds such as CTMB, which simultaneously target GABA_A receptors and voltage-gated sodium channels, have demonstrated reductions in neuronal injury and promotion of synaptic plasticity in TBI models, further supporting multi-channel modulation as a potential strategy [13].

Despite these encouraging results, significant translational challenges remain. Variability among animal models, including differences in injury severity, mechanism, and timing of intervention, limits direct extrapolation to human TBI [33]. In addition, physiological differences and the marked heterogeneity of injury patterns in clinical populations complicate the interpretation and application of experimental findings [11]. Although preclinical evidence provides a strong mechanistic foundation, well-designed clinical trials remain essential to determine the safety, tolerability, and real-world effectiveness of these agents [11]. Differences in mechanism also warrant consideration; for instance, memantine targets NMDA-mediated excitotoxicity, whereas nimodipine mitigates calcium influx and histone-induced neuronal injury through distinct pathways [2,33]. Understanding these distinctions may be critical in refining mechanism-guided therapeutic strategies.

Clinical considerations in the anesthetic management of acute TBI

Anesthetic and sedative selection in TBI requires careful balancing of hemodynamic stability with preservation of intracranial dynamics. ICP and cerebral perfusion pressure (CPP) remain central determinants of neurological outcome, and any pharmacologic strategy must prioritize their stability. Elevated ICP poses a major threat to cerebral perfusion, while hypotension can critically compromise CPP and worsen secondary injury.

Propofol and midazolam are widely used for sedation in TBI and demonstrate comparable overall efficacy and safety profiles. Both are generally considered acceptable with respect to ICP control; however, high doses of propofol may impair cerebral autoregulation, raising concerns in patients with already compromised perfusion [35]. In addition, propofol-induced hypotension may reduce CPP if not carefully titrated.

Dexmedetomidine has emerged as a valuable adjunct in neurocritical care, providing effective sedation and potential modulation of secondary injury pathways. Its sympatholytic properties may attenuate agitation and stress responses; however, its propensity to induce hypotension requires cautious use, particularly in patients with marginal CPP [36]. Careful hemodynamic monitoring is therefore essential when incorporating dexmedetomidine into sedation regimens.

Ketamine, historically avoided due to concerns about ICP elevation, is now supported by more recent evidence indicating that it does not significantly increase ICP and may reduce cortical spreading depolarizations [37]. Importantly, ketamine tends to preserve or even support CPP, reinforcing its utility in selected TBI patients, particularly those at risk of hypotension [37,38].

Opioids, although effective for analgesia, may increase ICP and reduce CPP, necessitating judicious dosing and close monitoring in neurocritical settings [36]. Combination regimens further complicate management. For example, the concurrent use of dexmedetomidine and propofol may amplify hypotensive effects, increasing the risk of compromised cerebral perfusion.

## Conclusions

Secondary brain injury after TBI represents a dynamic and potentially modifiable process driven by ionic imbalance, excitotoxicity, mitochondrial dysfunction, inflammation, blood-brain barrier disruption, and impaired cerebral autoregulation. Unlike the primary insult, this evolving phase offers a meaningful therapeutic window in which targeted interventions may attenuate neuronal loss and limit cerebral edema.

Ion-channel dysregulation, particularly involving calcium influx, sodium-mediated depolarization, and glutamatergic overactivation, emerges as a unifying mechanistic pathway in this cascade. Anesthetic agents with ion-channel-modulating properties, therefore, extend beyond their sedative function and may contribute to neuroprotection when integrated within a physiologically guided management strategy. Agents targeting NMDA receptors, voltage-gated sodium channels, and calcium channels show the strongest mechanistic and translational rationale, although clinical evidence remains heterogeneous.

A mechanism-guided approach to anesthetic selection, prioritizing intracranial stability, preservation of CPP, and modulation of dominant secondary injury pathways, may enhance neuroprotective strategies in both neuro-intensive care unit (ICU) and perioperative settings. Nevertheless, robust, high-quality clinical trials are essential to define which agents, dosing strategies, and clinical contexts provide meaningful outcome benefits in patients with acute TBI.
